# The Effect of Intrauterine Autologous Peripheral Blood Mononuclear Cells (PBMCs) Administration in Modulating the Immunologic Profile Aberrations in Repeated Implantation Failure (RIF) Women

**DOI:** 10.1002/rmb2.12686

**Published:** 2025-10-21

**Authors:** Samaneh Abdolmohammadi‐Vahid, Mohammad Sadegh Soltani‐Zangbar, Ali Aghebati‐Maleki, Narges Nouri, Hamid Ahmadi, Shahla Danaii, Leili Aghebati‐Maleki

**Affiliations:** ^1^ Immunology Research Center Tabriz University of Medical Sciences Tabriz Iran; ^2^ Department of Molecular Medicine, Faculty of Advanced Medical Sciences Tabriz University of Medical Sciences Tabriz Iran; ^3^ Department of Medical Biology and Central Electron Microscope Laboratory, Medical School P'ecs University P'ecs Hungary; ^4^ Gynecology Department Eastern Azerbaijan ACECR, ART Center Eastern Azerbaijan, Branch of ACECR Tabriz Iran; ^5^ Department of Immunology, School of Medicine Tabriz University of Medical Sciences Tabriz Iran

**Keywords:** implantation, PBMC, RIF, Th17, Treg

## Abstract

**Purpose:**

Considering the immune‐related etiology of RIF, administration of an immunomodulatory approach such as autologous PBMCs seems to be helpful in modifying the dysregulated immune responses.

**Methods:**

100 RIF women were divided into the PBMC receiving and the control group. Blood sampling was conducted 48 h before and 2 weeks after PBMC administration. The frequency of Th17, Treg, and NK cells, the expression level of related transcription factors and miRNAs, and the concentration of inflammatory and anti‐inflammatory cytokines were evaluated pre‐ and post‐treatment. Pregnancy outcome including pregnancy, live birth, and miscarriage rates were also evaluated.

**Results:**

PBMC therapy significantly elevated the frequency of Th17 and NK cells (*p* = 0.0035 and 0.0013, respectively) and the expression of RORγt (*p* < 0.0001), in comparison with pre‐treatment, while the frequency of Treg cells (*p* = 0.0063) and the expression of FoxP3 and PTEN were decreased post treatment. The serum concentration of IL‐1β, IL‐17, and TNF‐α was increased, while IL‐10 and TGF‐β were reduced post‐treatment when compared to pre‐treatment. Pregnancy and live birth rates were significantly higher in the PBMC‐treated group in comparison with routine treatment.

**Conclusion:**

Intrauterine administration of autologous PBMCs may be helpful in treating RIF patients, especially those with decreased inflammatory cells and mediators in the implantation process.

## Graphical abstract

The experiment procedure of the study.

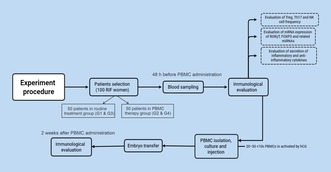

## 1 Introduction

Embryo implantation is the early stage of pregnancy in which the blastocyst attachment to the endometrial epithelium occurs in a coordinated event to support embryo growth [1]. A typical and healthy endometrium and bidirectional signaling between the endometrial epithelium and embryo are essential for a successful implantation [2]. Failure in implantation, which leads to embryo loss, makes the implantation process a critical limiting factor in the establishment of a successful pregnancy [3]. In vitro fertilization (IVF) is among the assisted reproductive technologies (ART) for improving fertility problems such as implantation failure [2, 4]. Although there has not been an agreement upon the definition of the condition known as recurrent implantation failure (RIF), failure in implantation and achieving pregnancy after transferring at least three good quality embryos in the IVF procedure is called RIF [1].

Solid evidence exists about the role of immune system imbalance in implantation failure [1, 5, 6]. Failure in maternal immunotolerance toward the embryo or fetus would cause pregnancy wastage. Regulatory mechanisms against the inflammatory cells and mediators are essential in the maintenance of a pregnancy [7]. On the other hand, an inflammatory microenvironment is required for uterus preparation in the early implantation stage [8]. Therefore, a delicate balance between T cell subsets is essential in the process of normal implantation and pregnancy, including a shift toward T helper 1 (Th1) cells and cytokines like interferon‐ɣ (IFNɣ) at the early stage of implantation and a shift toward Th2 cells and cytokines such as interleukin‐4 (IL‐4) and IL‐10 after implantation [9].

On the other hand, regulatory T cells (Treg) are the central mediator of tolerance in the feto‐maternal crosstalk and are involved in the implantation process and embryo development [10]. The involvement of Th17 inflammatory cells in human reproduction was indicated in the studies, which demonstrated that the increased frequency of these cells and associated cytokines, including IL‐17, was associated with a greater risk of pregnancy wastage [10, 11]. The ratio of Th17/Treg cells is among the predictors of the risk of pregnancy wastage; in addition to the Th1/Th2 ratio, failure in achieving the proper Th1/Th2 and Th17/Treg ratios is associated with reproductive failures such as RIF [12, 13]. Uterine natural killer (uNK) cells are the predominant population of the placenta in the early stage of pregnancy, which also play a crucial role in trophoblast invasion [14]. Data are accumulating that supports the role of immunologic dysregulation in the pathogenesis of implantation failure and pregnancy wastage [9, 11].

A group of endogenous short non‐coding RNAs, known as miRNAs, is the primary regulator of gene expression and various biological pathways, including hematopoiesis, inflammation, and the function of different immune cells by post‐transcriptional regulation of associated messenger RNAs (mRNAs) [15]. According to the literature, dysregulation of miRNA expression is involved in various pathologies, such as immune system‐related disorders [16]. In addition, miRNAs can regulate immune cell proliferation and function. For instance, Treg cell differentiation and function are controlled by mir‐155 by targeting forkhead box P3 (FoxP3), the main transcription factor of Treg. Decreased expression of this miRNA can affect the frequency of Treg cells [17, 18]. Mir‐155 also targets phosphatase and tensin homolog (PTEN), a tumor suppressor gene and a protein/lipid phosphatase, which may be involved in mice's decidualization and trophoblastic cell invasion [19]. In addition to mir‐155, mir‐940, which is downregulated in RIF patients, can also impair the proliferation of trophoblast cells. The essential proteins, including nuclear factor kappa‐light‐chain‐enhancer of activated B cells (NF‐κB), Wnt/β‐catenin, and zinc finger protein 672 (ZNF24), are the proposed targets of mir‐940 [20]; nevertheless, it is postulated that mir‐940 impairs the proliferation of trophoblast cells and promotes pregnancy wastage by targeting ZNF24 [21]. Dickkopf WNT Signaling Pathway Inhibitor 1 (DKK1) is also targeted by mir‐940 and mir‐144, downregulated in RIF women [22]. DKK1 can affect the implantation process by regulating embryo attachment [23]. On the other hand, cyclooxygenase‐2 (Cox‐2), as a target of mir‐144‐3p, is negatively regulated by this miRNA, as well as several inflammatory mediators, including IL‐1β, IL‐6, and tumor necrosis factor‐alpha (TNF‐α) [24].

The transforming growth factor‐beta (TGF‐β) pathway is essential for Treg cells' differentiation and function, modulated by proapoptotic gene activity, called BCL2L11/Bim [25]. Bim is the target gene of mir‐10a, and mir‐10a modulates cell apoptosis by downregulating the proapoptotic protein Bim [26]. Therefore, mir‐10a is the other regulator of Treg cells. A substitution polymorphism in miR‐10a may lead to the attenuated sensibility of cells to progesterone and pregnancy wastage. Target analysis of mir‐20b‐5p, a member of the mir‐106a‐363 cluster, indicated that upregulation of this miRNA could downregulate the expression of PTEN and Bim [27]. Mir‐718 is an anti‐inflammatory miRNA that targets Interleukin‐1 receptor‐associated kinase 1 (IRAK1) and subsequently negatively regulates NF‐κB activation, a transcription regulator of inflammatory cytokines. A systemic analysis of the miRNA profile of RIF women indicated that the expression of mir‐718 is downregulated in RIF women [23].

Given all the above, there is accumulating data indicating the role of immune cells and related miRNA in the pathogenesis of immune‐related reproductive disorders; therefore, an immunomodulatory approach is required for modifying the dysregulated immune responses through the implantation process [12]. Indeed, modulation of the maternal immune system in the case of immunologic abnormalities by immunologic approaches may help achieve or maintain the pregnancy and successful outcomes. Intrauterine peripheral blood mononuclear cells (PBMCs) therapy for reproductive failures is among these approaches [28]. Administration of PBMCs, containing T and B lymphocytes and monocytes, is capable of improving the implantation rate in RIF patients and positively affecting the blastocyte invasion to endometrium by upregulation of matrix metalloproteinase‐2 (MMP‐2) and MMP‐9 expression [29]. In addition, immunologic tolerance toward the embryo is improved following the intrauterine PBMCs administration [30], and PBMCs regulate the secretion of inflammatory cytokines such as IL‐1β and TNF‐α [31].

The current study aims to investigate the effect of intrauterine PBMCs administration on modulating RIF patients' immunologic abnormalities. To our knowledge, this is the first study in which the effect of intrauterine administration of autologous PBMCs was investigated on immunologic cells such as Th17, Treg, and NK cells, related cytokines, transcription factors, and miRNAs in humans, pre‐ and post‐treatment.

## 2 Material and Methods

### 2.1 Study Design and Subjects' Selection

Initially, one hundred RIF women were enrolled in the study. The participants were divided into two groups: 50 RIF patients who received intrauterine injections of human chorionic gonadotrophin (hCG)‐activated autologous PBMCs, 2 days before embryo transfer and 50 RIF patients who received routine treatment for RIF patients before ET. The immunologic parameters have been evaluated before (G2) and after (G4) PBMCs therapy and compared with the results of immunologic evaluations of women before (G1) and after (G3) routine treatment.

All subjects were under the following inclusion criteria: age between 20 and 45, three or more previous failures of in vitro fertilization‐ embryo transfer (IVF‐ET) therapy, and regular menstrual cycles. In the case of ovulatory disorder, anomalies, chromosomal aberrations, malignancies, diabetes, hypertension, infection, and other underlying diseases or receiving steroids or immunosuppressive drugs, the patients were excluded from the study. In addition, a semen analysis and DNA fragmentation test were performed for the partners of patients to select the patients with partners with typical test results. Demographic data of subjects is summarized in Table 1.

**TABLE 1:** Clinical findings of the RIF patients.

Variable	PBMCs receiving group mean ± SD (*N* = 50)		Control group mean ± SD (*N* = 50)		*p*	
Maternal age (years, range)	32.89 ± 3.31 (23–42)		33.46 ± 3.44 (25–43)		NS	
Body mass index (kg/m^2^)	26.54 ± 2.68		27.73 ± 3.11		NS	
Duration of infertility (years, range)	8.68 (4–18)		9.12 (3–20)		NS	
Number of transferred embryos	2.7 ± 1.32		2.93 ± 1.4		NS	
No. of smoking patient	2		0		NS	
No. of smoking partners	19		23		NS	
No. of previous ET attempts	4.1 ± 2.03		4.4 ± 2.41		NS	
No. of fresh cycles ET	22		21		NS	
Abnormal karyotype	0		0		NS	
Male infertility	0		0		NS	
FSH (mIU/l)	6.71 ± 2.63		7.11 ± 2.94		NS	
LH (mIL/l)	4.49 ± 1.8		4.56 ± 1.54		NS	
E2 (pg/ml)	36.16 ± 14.31		35.21 ± 13.86		NS	
PRL (ng/ml)	14.02 ± 5.12		12.35 ± 5.88		NS	
Anti‐TPO	0		0		NS	
Anti‐TG	0		0		NS	
APA	0		0		NS	
ACA	0		0		NS	
ANA	0		0		NS	
Anti‐ds DNA	0		0		NS	
Pregnancy rate	23 (46%)		12 (24%)		0.035	
Misscarriage rate	Total	From pregnant cases	Total	From pregnant cases	Total	From pregnant cases
	6/50 (12%)	6/23 (26%)	5/50 (10%)	5/12 (41.66%)	NS	0.18
Live birth rate	17/50 (34%)	17/23 (73.91%)	7/50 (14%)	7/12 (58.33%)	0.034	NS

Abbreviations: ACA, anti‐cardiolipin antibody; ANA, anti‐nuclear antibody; Anti‐ds DNA, anti‐double strand DNA; Anti‐TG, anti‐thyroglobulin; Anti‐TPO, anti‐thyroid peroxidase; APA, anti‐phospholipid antibody; BMI, body mass index; E2, estradiol 2; FSH, follicle stimulating hormone; HDL, high‐density lipoprotein; LDL, low‐density lipoprotein; LH, luteinizing hormone; PRL, prolactin.

Written informed consent was obtained from all the participants, and the study protocol was approved by the Research Ethics Committee of Tabriz University of Medical Sciences (IR. IR.NIMAD.REC.1400.4002376).

The first blood sampling was conducted 48 h before PBMC administration. The second was done 2 weeks after PBMC administration in both PBMC receiving and control groups to evaluate and compare the immunologic parameters pre‐ and post‐treatment.

### 2.2 PBMC Isolation and Injection

For PBMCs isolation, 20 mL of peripheral blood of each participant was collected in heparinized tubes at the time of ovulation induction. Gradient centrifugation using Ficoll‐Paque PLUS (GE Healthcare Bio‐Sciences AB) was utilized for PBMCs isolation. After centrifugation for 20 min at 450 g of the tube containing blood overlayed on Ficoll, PBMCs were collected from the interphase layer. The collected cells were washed twice with Roswell Park Memorial Institute 1640 (RPMI 1640) medium (Gibco BRL Co. Ltd., Grand Island, NY, USA), followed by suspension of 20–30 × 10^6^ PBMCs in 8 mL RPMI 1640 supplemented with 10% heat‐inactivated FBS, and incubated in the presence of hCG (10 IU/mL daily) for 48 h at 37°C and 5% CO2 in 95% humidity incubator. After washing, 15 × 10^6^ PBMCs were suspended in 500 μL PBS, followed by gentle administration of PBMCs to the uterine cavity using an embryo transfer catheter, which occurs 2 days before ET.

### 2.3 Evaluation of Treg, Th17, and NK Cells Frequency

Flow cytometry technique was used to analyze the frequency of Th17, Treg, and NK cells, prior to and post‐PBMC therapy. Initially, the cells (1 × 10^6^) were stained with monoclonal antibodies against the surface markers, including fluorescein isothiocyanate (FITC)‐conjugated anti‐human CD4 (Clone SK3, 20 μL per test, BD Biosciences) for both Treg and Th17 cells. For evaluation of Treg cells frequency, phycoerythrin (PE)‐conjugated anti‐human CD25 (Clone 2A3 (RUO (GMP)), 20 μL per test, BD Biosciences) and allophycocyanin (APC)‐conjugated anti‐human CD127 (Clone HIL‐7R‐M21 (RUO), 5 μL per test, BD Biosciences) were also added to cells. Afterward, the cells were incubated at 4°C for 30 min, and then the cells were washed twice with phosphate‐buffered saline (PBS) (Sigma, Germany) and analyzed using FACS Calibur (BD Biosciences) flow cytometer. To evaluate Th17 cell frequency, the cells were stimulated with 10 ng/mL of PMA and 0.5 μM ionomycin (eBioscience) for 5 h at 37°C in a 5% CO2 incubator. In the next step, 0.5 μM of monensin (eBioscience) was added to cells to inhibit cytokine secretion and improve intracellular staining. After incubation time, the cells were collected by centrifugation at 300 g for 10 min. Afterward, the cells were prepared for intracellular staining using fixation and permeabilization buffer (eBioscience) and incubated at room temperature for 20 min. In the next step, PE‐conjugated anti‐human IL‐17 (Clone N49‐653 (RUO), 20 μL per test, BD Biosciences) was added and incubated for 30 min at room temperature. Afterward, the cells were washed and were ready for analysis.

Anti‐CD3 (Clone SK7 (also known as Leu‐4), 20 μL per test, BD Biosciences), Anti‐CD16 (Clone 3G8 (RUO) 20 μL per test, BD Biosciences), and anti‐CD56 (Clone NCAM16.2 (also known as NCAM 16), 5 μL per test, BD Biosciences) antibodies labeled with FITC, PE, and APC fluorochromes, respectively, were used for surface staining of NK cells. The FACS Calibur flow cytometer (BD Biosciences) and FlowJo software (Becton Dickinson, Mountain View, CA, USA) were used to analyze the population, and 10000 events were counted for each sample. Ultimately, the population of CD4^+^CD25^+^CD127^−/low^ cells was determined as Treg cells, IL‐17^+^CD4^+^ cells were defined as Th17 cells, and CD3^+^CD16^+^CD56^+^ cells were determined as NK cells.

### 2.4 Evaluation of mRNA Expression Level of miRNAs, Transcription Factors, and PTEN

The gene expression of FoxP3 and RORγt, Treg, and Th17 associated transcription factors, alongside the gene expression of PTEN and mir‐10a, mir‐20b‐5p, mir‐144‐3p, mir‐155, mir‐718, and mir‐940, was evaluated among isolated PBMCs of RIF patients, before and after treatment, by using the real‐time polymerase chain reaction (PCR) method. TRIzol reagent (Sigma‐Aldrich, Steinheim, Germany) was utilized to extract the total RNA of isolated PBMCs, according to the manufacturer's recommendations. Extracted RNA was used as a template for cDNA synthesis with the help of the Revert Aid reverse transcriptase and random hexamer primer (Thermo Fisher Scientific).

SYBR Green method and the LightCycler 2.0 Real‐Time PCR System machine (Roche Applied Sciences, Mannheim, Germany) were utilized to evaluate gene expression using specific primers listed in Table 2. The thermocycling process was as follows: 95°C for 10 s (DNA denaturation) repeated for 40 cycles, 60°C for 10 s (annealing step), and 72°C for 20 s (extension step). Using the 2^−ΔΔCt^ formula, the comparative Ct method was used to calculate the relative expression of target genes compared to the housekeeping controls, β‐actin and U6.

**TABLE 2:** Primer pair sequences.

Gene	Primer	Sequence (5′ → 3′)
*RORγT*	Forward	GAGGAAGTGACTGGCTACCAGA
	Reverse	GCACAATCTGGTCATTCTGGCAG
*FoxP3*	Forward	GGCACAATGTCTCCTCCAGAGA
	Reverse	CAGATGAAGCCTTGGTCAGTGC
*PTEN*	Forward	TGAGTTCCCTCAGCCGTTACCT
	Reverse	GAGGTTTCCTCTGGTCCTGGTA
*miR‐10a*	Forward	CCTGTAGATCCGAATTTG
	Reverse	GAACATGTCTGCGTATCTC
*miR‐20b‐5p*	Forward	TGCTCATAGTGCAGGTAG
	Reverse	GAACATGTCTGCGTATCTC
*miR‐144‐3p*	Forward	GGATATCATCATATACTGTA
	Reverse	GAACATGTCTGCGTATCTC
*miR‐155*	Forward	TGCTAATCGTGATAGGGG
	Reverse	GAACATGTCTGCGTATCTC
*miR‐718*	Forward	CTTCCGCCCCGCCGGGC
	Reverse	GAACATGTCTGCGTATCTC
*miR‐940*	Forward	AAGGCAGGGCCCCCG
	Reverse	GAACATGTCTGCGTATCTC
*β‐Actin*	Forward	CACCATTGGCAATGAGCGGTTC
	Reverse	AGGTCTTTGCGGATGTCCACGT
*U6*	Forward	CTCGCTTCGGCAGCACAT
	Reverse	TTTGCGTGTCATCCTTGCG

Abbreviations: FoxP3: forkhead box P3; miRs: micro RNAs; PTEN: phosphatase and TENsin homolog; RORγT: retinoic acid‐related orphan receptor gamma t.

### 2.5 Evaluation of Inflammatory and Anti‐Inflammatory Cytokines

The secretion levels of inflammatory (IL‐1β, TNF‐α, and IL‐17) and anti‐inflammatory (TGF‐β, IL‐10) cytokines (pg/mL) were measured in the serum of RIF patients, pre and post‐PBMC therapy, employing the ELISA kit (MyBioSource, San Diego, CA), based on the manufacturer's instructions. To enhance the accuracy of the test, all samples were analyzed in duplicate. A microplate reader measured the absorbance according to the standard curve.

### 2.6 Statistical Analysis

In the current study, the SPSS PC Statistics Software (version 19.0; SPSS Inc., Chicago, IL) and GraphPad Prism (version 7.00 for Windows; GraphPad Software, La Jolla, CA) were utilized to perform the statistical analysis and draw the graphs, respectively. Brown‐Forsythe ANOVA, followed by Dunnett's T3 multiple comparisons test, was employed to analyze the statistical differences in immunologic parameters between the groups (G1–G4). The data were indicated as the mean ± SD. *p* < 0.05 was represented as statistically significant. Pregnancy, live birth, and miscarriage rates were analyzed using Fisher's exact test among the study group.

## 3 Results

### 3.1 Circulating Treg, Th17, and NK Cells Frequency in RIF Patients, Pre‐ and Post‐Treatment

**FIGURE 1:**
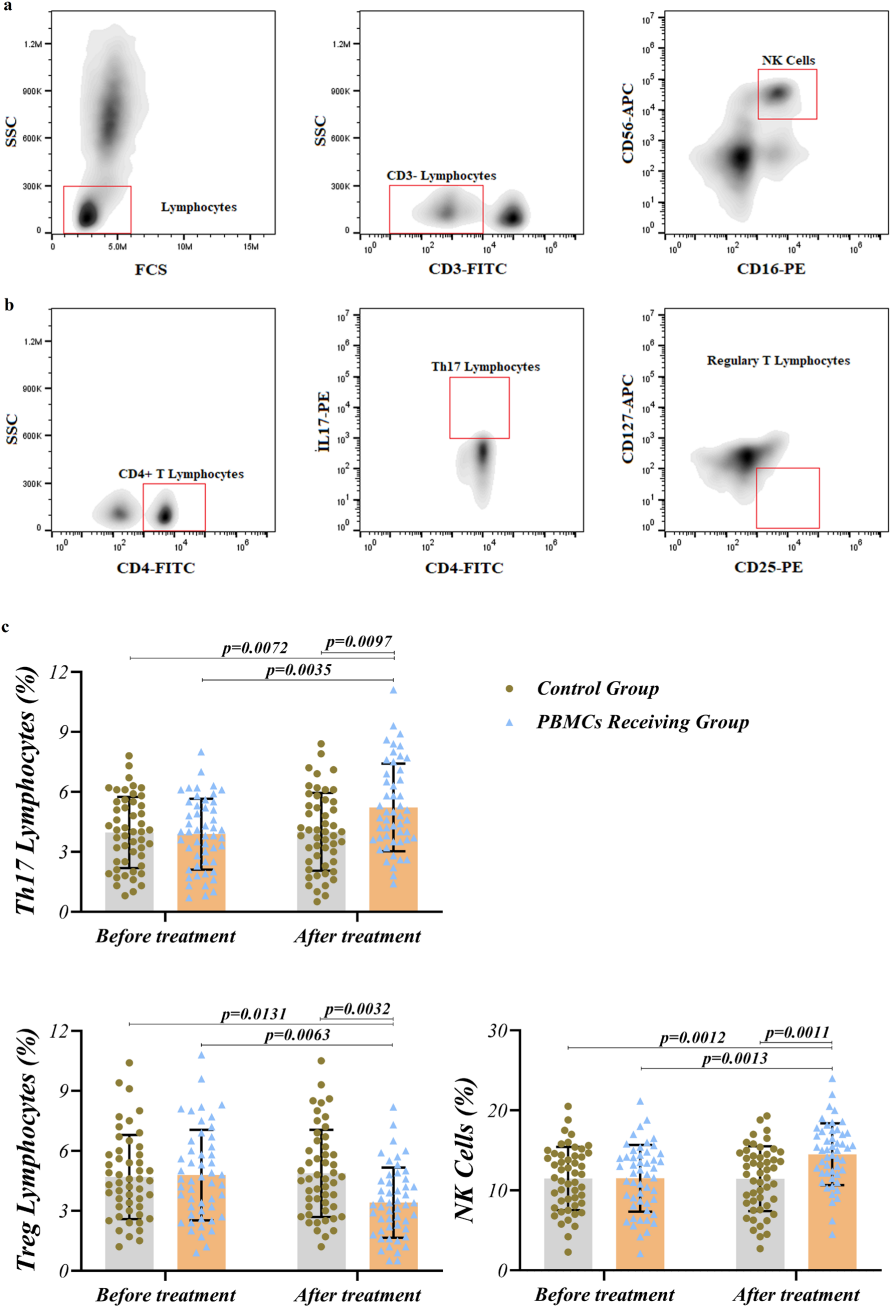
Treg, Th17, and NK cell frequency in RIF patients pre‐ and post‐PBMC therapy. Panel (a, b) illustrates the gating strategy and the abundance of Tregs, Th17 cells, and NK cells. (c) PBMC therapy can significantly upregulate Th17 (*p* = 0.0035) and NK cells (*p* = 0.0013) frequency in RIF patients, compared with pre‐treatment. In contrast, when compared with pre‐treatment, Treg cells were decreased due to PBMCs administration in RIF women (*p* = 0.0063). PBMCs administration also significantly elevated the frequency of Th17 (*p* = 0.097) and NK cells (*p* = 0.0011) compared with routine treatment. In addition, the frequency of Treg cells was also significantly affected by PBMCs therapy, as it significantly decreased in PBMC‐receiving RIF patients compared to the routine treatment group (*p* = 0.0032). A difference was considered significant when *p* < 0.05 (PBMC‐receiving RIF patients; *n* = 50, Control group; *n* = 50).

The results of our study indicated that there are no statistically significant differences in the frequency of Treg, Th17, and NK cells before and after routine treatment in the RIF group (G1 vs. G3) and among PBMCs receiving and routine treatment group before the treatment (G2 vs. G1) (Table 3). At the same time, PBMC therapy can significantly upregulate Th17 (*p* = 0.0035) and NK cells (*p* = 0.0013) frequency in these patients (G4), in comparison with pre‐treatment (G2). However, Treg cells were decreased as a result of PBMCs administration in RIF women (4.79% ± 2.26%) when compared with pre‐treatment (3.414% ± 1.755%) (*p* = 0.0063). Figure 1a,b illustrates the gating method and the abundance of Tregs, Th17 cells, and NK cells.

**TABLE 3:** Summary of molecular and immunological characteristics of RIF patients before and after receiving PBMCs.

Target	RIF patients before routine treatment (*N* = 50) mean ± SD (G1)	RIF patients before PBMCs receiving (*N* = 50) mean ± SD (G2)	RIF patients after routine treatment (*N* = 50) mean ± SD (G3)	RIF patients after PBMCs receiving (*N* = 50) mean ± SD (G4)	*p* value G1 vs. G2	*p* value G1 vs. G3	*p* value G1 vs. G4	*p* value G2 vs. G3	*p* value G2 vs. G4	*p* value G3 vs. G4
**Flow cytometry**										
Th17 (%)	3.946 ± 1.781	3.88 ± 1.771	4 ± 1.949	5.222 ± 2.194	NS	NS	0.0072	NS	0.0035	0.097
Treg (%)	4.692 ± 2.106	4.79 ± 2.26	4.874 ± 2.181	3.414 ± 1.755	NS	NS	0.0131	NS	0.0063	0.0032
NK cells (%)	11.492 ± 3.953	11.502 ± 4.174	11.466 ± 4.054	14.516 ± 3.865	NS	NS	0.0012	NS	0.0013	0.0011
**Relative gene expression (fold change)**										
*RORγT*	1.000 ± 0.071	0.99 ± 0.294	1.071 ± 0.476	1.43 ± 0.722	NS	NS	< 0.0001	NS	< 0.0001	0.0007
*FoxP3*	1.000 ± 0.097	1.024 ± 0.55	0.933 ± 0.313	0.72 ± 0.283	NS	NS	0.0005	NS	0.0001	0.0139
*PTEN*	1.000 ± 0.084	0.983 ± 0.535	0.955 ± 0.381	0.697 ± 0.311	NS	NS	0.0003	NS	0.0007	0.0029
*miR‐10a*	1.000 ± 0.062	1.019 ± 0.247	0.961 ± 0.408	0.676 ± 0.204	NS	NS	< 0.0001	NS	< 0.0001	< 0.0001
*miR‐20b*	1.000 ± 0.076	1.021 ± 0.372	0.95 ± 0.26	0.713 ± 0.602	NS	NS	0.0012	NS	0.0004	0.0109
*miR‐144*	1.000 ± 0.08	0.977 ± 0.217	1.044 ± 0.45	1.762 ± 1.623	NS	NS	< 0.0001	NS	< 0.0001	0.0002
*miR‐155*	1.000 ± 0.077	1.017 ± 0.218	1.08 ± 0.519	1.663 ± 1.395	NS	NS	0.0001	NS	0.0002	0.0009
*miR‐718*	1.000 ± 0.102	0.97 ± 0.453	1.037 ± 0.405	1.578 ± 1.284	NS	NS	0.0004	NS	0.0002	0.0011
*miR‐940*	1.000 ± 0.094	0.989 ± 0.408	0.936 ± 0.306	2.146 ± 1.53	NS	NS	< 0.0001	NS	< 0.0001	< 0.0001
**ELISA (serum)**										
IL‐17 (pg/ml)	12.42 ± 6.719	13.33 ± 7.042	12.82 ± 6.134	17.47 ± 9.052	NS	NS	0.0038	NS	0.0264	0.0093
IL‐1β (pg/ml)	6.346 ± 5.265	6.774 ± 5.396	6.896 ± 6.229	11.656 ± 9.394	NS	NS	0.0007	NS	0.0023	0.0031
TNF‐α (pg/ml)	7.3 ± 6.602	6.942 ± 5.648	7.198 ± 6.093	11.222 ± 7.394	NS	NS	0.0145	NS	0.0061	0.0114
IL‐10 (pg/ml)	5.162 ± 2.699	5.016 ± 2.516	5.388 ± 2.509	3.734 ± 1.789	NS	NS	0.0175	NS	0.0410	0.0039
TGF‐β (pg/ml)	40.84 ± 20.975	42.02 ± 21.58	41 ± 24.108	28.76 ± 15.594	NS	NS	0.0212	NS	0.0090	0.0190

*Note:* Data are presented as mean ± standard deviation. *p* < 0.05 is considered as significant.

Abbreviations: FoxP3, Forkhead box P3; ILs, Interleukins; miRs, micro RNAs; NK cells, natural killer cells; PTEN, phosphatase and TENsin homolog; RIF, repeated implantation failure; RORγT, retinoic acid‐related orphan receptor gamma t; TGF‐β, transforming growth factor beta; Th17, T helper 17 lymphocytes; TNF‐α, tumor necrosis factor alpha; Treg, regulatory T lymphocytes.

The results also showed significant differences in the frequency of immune cells after routine treatment protocol for RIF women and intrauterine PBMC therapy. PBMCs administration was able to significantly elevate the frequency of NK cells (*p* = 0.0011) when compared with routine treatment (G3) (Figure 1c). Although the population of Th17 cells was elevated by PBMC therapy in comparison with the routine treatment group, this increase was not significant. In addition, the frequency of Treg cells was also significantly affected by PBMCs therapy (G4), as it was significantly decreased (*p* = 0.0032) in PBMC‐receiving RIF patients when compared to the routine treatment group (G3) (Figure 1c). The results are presented in detail in Table 3.

### 3.2 Transcription Level of miRNAs, Transcription Factors, and PTEN in RIF Patients, Pre‐ and Post‐Treatment

**FIGURE 2:**
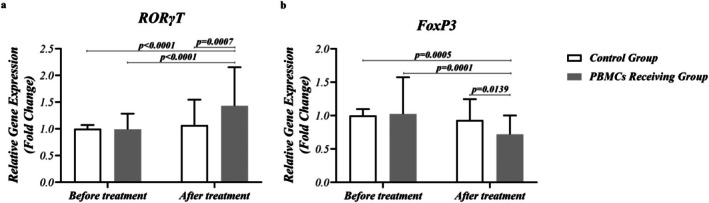
Expression level of RORɣt and FoxP3 in RIF patients, pre‐ and post‐PBMC therapy. (a) The expression level of RORɣt was upregulated following PBMC therapy in RIF patients, compared with the pre‐treatment level (*p* < 0.0001). In addition, PBMC therapy succeeded in increasing the mRNA expression level of RORɣt, compared with routine treatment protocol (*p* = 0.0007). (b) In the case of FoxP3, a noticeable decrease was observed after treatment with PBMCs, compared with pre‐treatment (*p* = 0.0001). Additionally, the downregulation of Foxp3 expression was higher in the PBMC‐receiving group compared with the routine treatment group (*p* = 0.0139). A difference was considered significant when *p* < 0.05 (PBMC‐receiving RIF patients; *n* = 50, Control group; *n* = 50).

The transcription level of Treg and Th17‐related transcription factors, FoxP3 and RORγt, was evaluated in PBMCs of RIF patients in G1–G4 groups. The results demonstrated that the expression level of RORγt was upregulated as a result of PBMC therapy in RIF patients (G4), compared with the pre‐treatment level (G2) (*p* < 0.0001) (Figure 2a). However, the routine treatment protocol could not significantly affect the transcription level of RORγt (G1 vs. G3). In addition, PBMC therapy was successful in increasing the mRNA expression level of RORγt, in comparison with the routine treatment protocol (G3 vs. G4) (*p* = 0.0007). Figure 2 illustrates RORγT and FoxP3 gene expression in PBMCs and routine treatment‐receiving groups before and after treatment.

**FIGURE 3:**
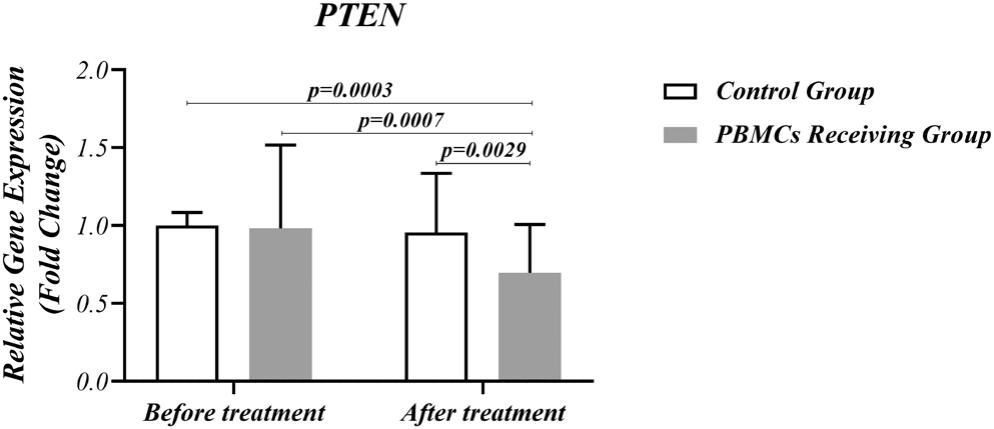
Expression level of PTEN in RIF patients, pre‐ and post‐PBMC therapy. An apparent decrease was observed in PTEN mRNA expression after treatment with PBMCs, compared with pre‐treatment (*p* = 0.0007). Additionally, the downregulation of PTEN expression was higher in the PBMC‐receiving group than in the routine treatment group (*p* = 0.0029). A difference was considered significant when *p* < 0.05 (PBMC‐receiving RIF patients; *n* = 50, Control group; *n* = 50).

Figure 3 illustrates PTEN gene expression in PBMCs and routine treatment–receiving groups before and after treatment. In the case of FoxP3 and PTEN, a noticeable decrease was observed after treatment with PBMC (G4), compared with pre‐treatment (G2) (*p* = 0.0001 and *p* = 0.0007, respectively). Additionally, the downregulation of Foxp3 and PTEN expression was higher in the G4 group, compared with G3 (*p* = 0.0139 and *p* = 0.0029, respectively) (Figures 2b and 3). There was no significant difference regarding the expression of RORγt, Foxp3, and PTEN between PBMCs–receiving and routine treatment groups before the treatment (G2 vs. G1) (Figures 2b and 3 and Table 3).

**FIGURE 4:**
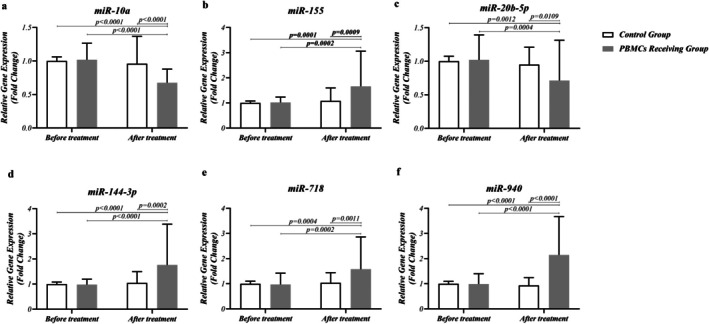
Expression level of miRNAs in RIF patients, pre‐and post‐PBMC therapy. (a, c) As a result of PBMC therapy in RIF patients, the expression of mir‐10a and mir‐20b was downregulated in the PBMC receiving group, compared with the routine treatment group (*p* < 0.0001 and *p* = 0.0004, respectively). In contrast, PBMC therapy upregulates the expression of mir‐144 (d), mir‐155 (b), mir‐718 (e), and mir‐940 (f) post‐treatment (*p* < 0.0001, 0.0002, 0.0002, and < 0.0001, respectively). However, the routine treatment protocol could not significantly increase or decrease the expression of these miRNAs. In a comparison of the PBMC receiving group and routine treatment group, the observed decrease in mir‐10a and mir‐20b expression by PBMC therapy was more powerful than routine treatment protocol (*p* < 0.0001 and 0.0109, respectively), as the same was observed in downregulation of mir‐144, mir‐155, mir‐718 and mir‐940 (*p* = 0.0002, 0.0009, 0.0011 and < 0.0001, respectively). A difference was considered significant when *p* < 0.05 (PBMC‐receiving RIF patients; *n* = 50, Control group; *n* = 50).

Figure 4 illustrates the expression of different miRNAs in PBMCs and routine treatment‐receiving groups before and after treatment. As a result of PBMC therapy in RIF patients, the expression of mir‐10a and mir‐20b was downregulated in the G4 group, compared with the G2 group (*p* < 0.0001 and *p* = 0.0004, respectively) (Figure 4a,c). In contrast, PBMC therapy upregulates the expression of mir‐144, mir‐155, mir‐718, and mir‐940 post‐treatment (*p* < 0.0001, 0.0002, 0.0002, and < 0.0001, respectively) (Figure 4b,d–f). However, the routine treatment protocol could not significantly increase or decrease the expression of these miRNAs (G1 vs. G3).

In comparison of the G3 and G4 groups, the observed decrease in mir‐10a and mir‐20b expression by PBMC therapy was more powerful than the routine treatment protocol (*p* < 0.0001 and 0.0109, respectively), as the same was observed in the downregulation of mir‐144, mir‐155, mir‐718, and mir‐940 (*p* = 0.0002, 0.0009, 0.0011, and < 0.0001, respectively) (Table 3 and Figure 4b,d–f). No significant difference was observed regarding the expression of the mentioned miRNAs between the PBMCs‐receiving and routine treatment groups before the treatment (G2 vs. G1) (Figure 4 and Table 3).

### 3.3 Concentration of Inflammatory and Anti‐Inflammatory Cytokines in RIF Patients, Pre‐ and Post‐Treatment

**FIGURE 5:**
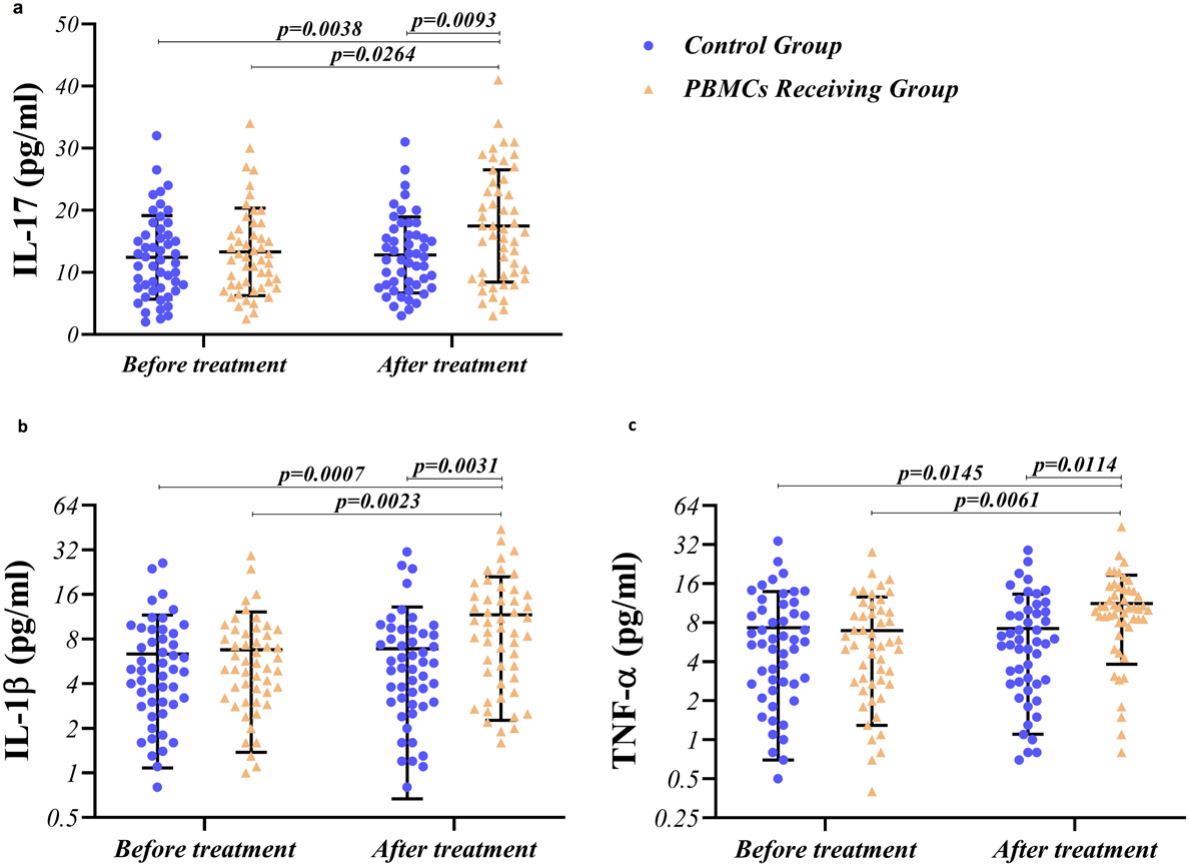
Concentration of inflammatory cytokines in RIF patients, pre‐ and post‐PBMC therapy. PBMC therapy could upregulate the concentration of inflammatory cytokines post‐PBMC therapy compared with the concentration of IL‐17 (a), IL‐1β (b), and TNF‐α (c) before the treatment (*p* = 0.0023, 0.0264, and 0.0061, respectively). Compared to PBMC therapy and routine treatment protocol, the concentration of abovementioned cytokines was also significantly higher in the PBMC therapy group (*p* = 0.0031, 0.0093, and 0.0114). A difference was considered significant when *p* < 0.05 (PBMC‐receiving RIF patients; *n* = 50, Control group; *n* = 50).

The concentration of inflammatory cytokines, including IL‐1β, IL‐17, and TNF‐α, was evaluated in the serum of RIF patients pre‐ and post‐PBMC therapy and routine treatment by ELISA. Figure 5 illustrates serum concentrations of inflammatory cytokines in PBMCs and routine treatment–receiving groups before and after treatment. Our results indicated that PBMC therapy was capable of upregulating the concentration of inflammatory cytokines post‐PBMC therapy when compared with the concentration of IL‐17 (Figure 5a), IL‐1β (Figure 5b), and TNF‐α (Figure 5c) before the treatment (G2 vs. G4) (*p* = 0.0023, 0.0264, and 0.0061, respectively). In comparing the G3 and G4 groups, the concentration of the abovementioned cytokines was also significantly higher in the G4 group (*p* = 0.0031, 0.0093, and 0.0114), as shown in Figure 5 and Table 3. However, no significant difference was observed regarding the concentration of these inflammatory cytokines between PBMCs–receiving and routine treatment groups before the treatment (G2 vs. G1) (Figure 5 and Table 3).

**FIGURE 6:**
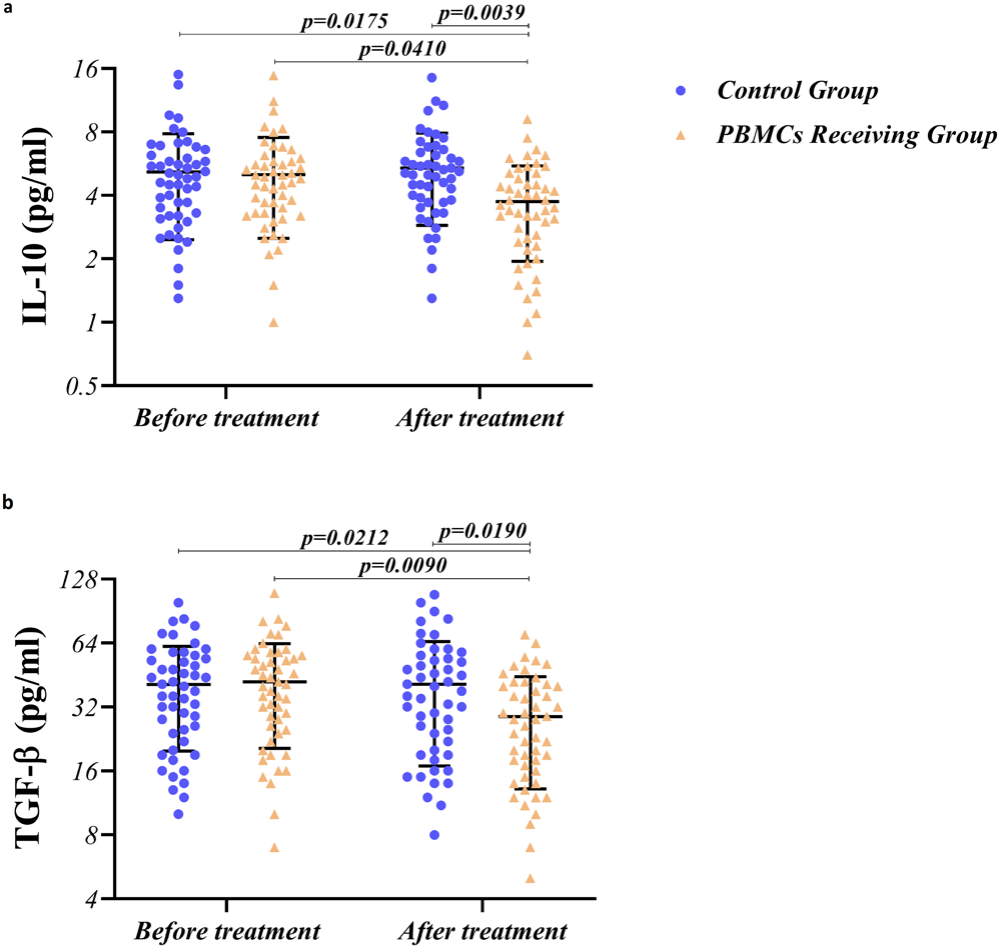
Concentration of anti‐inflammatory cytokines in RIF patients, pre‐ and post‐PBMC therapy. Intrauterine PBMC administration decreased the concentration of IL‐10 (a) and TGF‐β (b) cytokines post‐treatment compared to pre‐treatment (*p* = 0.0410 and 0.0090, respectively) in RIF patients. However, the routine treatment protocol did not decrease the secretion of IL‐10 and TGF‐β. PBMC therapy was also more potent in downregulating the production of IL‐10 and TGF‐β compared to routine treatment (*p* = 0.0039 and 0.0190, respectively). A difference was considered significant when *p* < 0.05 (PBMC‐receiving RIF patients; *n* = 50, Control group; *n* = 50).

Figure 6 illustrates serum concentrations of anti‐inflammatory cytokines in PBMCs and routine treatment‐receiving groups before and after treatment. In the case of anti‐inflammatory cytokines, including IL‐10 (Figure 6a) and TGF‐β (Figure 6b), intrauterine PBMC administration decreased the concentration of these cytokines post‐treatment (G4) when compared to pre‐treatment (*p* = 0.0410 and 0.0090, respectively) in RIF patients; however, the routine treatment protocol did not decrease the secretion of IL‐10 and TGF‐β (G1 vs. G3). As shown in Figure 6, PBMC therapy was more potent in downregulating the production of IL‐10 and TGF‐β in comparison with routine treatment (G3 vs. G4) (*p* = 0.0039 and 0.0190, respectively) (Table 3). There was no significant difference regarding the concentration of these anti‐inflammatory cytokines between PBMCs‐receiving and routine treatment groups before the treatment (G2 vs. G1) (Figure 6 and Table 3).

### 3.4 Pregnancy, Live Birth and Miscarriage Rate in PBMC Treated and Routine Treatment RIF Patients

The rate of pregnancy was significantly higher in PBMC‐treated RIF women (46%) when compared to that of the routine treatment group (24%, *p* = 0.035). PBMC therapy was capable of increasing the live birth rate in comparison with routine treatment protocols [17/50 (34%) vs. 7/50 (14%), *p* = 0.034], while there was no statistically significant difference among the groups regarding miscarriage rate in spite of the higher rate of miscarriage in the routine treatment group (Table 1).

## 4 Discussion

There is solid evidence about the role of immunologic aberrations in the pathogenesis of implantation failure; therefore, there is a growing need for immunomodulatory approaches in the case of immunologic disturbances' involvement in the pathogenesis of reproductive failure. Our study aimed to investigate the effect of intrauterine hCG‐activated PBMCs injection in improving immunologic abnormalities presented in RIF patients, with immunologic abnormalities including decreased peripheral Th17 and NK cell numbers and concentration of inflammatory cytokines (IL‐17, IL‐1β, and TNF‐α) and increased Treg frequency and concentration of IL‐10 and TGF‐β. According to our results, PBMC therapy, as a helpful approach, can increase the frequency of inflammatory Th17 cells alongside NK cells in peripheral blood. At the same time, this treatment protocol could downregulate the frequency of Treg cells in these patients. In line with these results, the gene expression level of related transcription factors was also affected by PBMC therapy in peripheral blood, as the expression level of RORγt, a Th17‐associated transcription factor, was increased post‐treatment.

In contrast, mRNA expression of FoxP3, a transcription factor of Treg cells, and PTEN expression was decreased. RIF patients who received the routine treatment protocol did not indicate any significant difference in the immunologic parameters before and after treatment. In addition, a comparison of the treatment results of PBMC receiving and routine treatment groups also demonstrated that PBMC therapy is more potent in the modulation of immunologic aberration in RIF patients.

At the same time, evaluation of inflammatory cytokines in peripheral blood, including IL‐17, IL‐1β, and TNF‐α, in the serum of RIF women in both groups demonstrated that the concentration of these cytokines is increased following treatment with PBMCs. In contrast, the concentration of regulatory cytokines, IL‐10 and TGF‐β, was downregulated post‐treatment. In summary, PBMC therapy shifted toward inflammatory responses, which are essential in implantation.

Additionally, evaluation of pregnancy outcome among the groups of the current study demonstrated that PBMC therapy was capable of improving the pregnancy and live birth rate and reducing the miscarriage rate among RIF women when compared with the RIF women who received routine treatment.

Alteration of the immune system to accept the semi‐allogenic developing embryo and fetus occurs at both local (uterine and decidua) and systemic (peripheral blood) levels, under the influence of steroid hormones. Since the immune cells in the decidua are directly in contact with the developing fetus and under the influence of an increased concentration of steroid hormone in female reproductive tracts, they undergo drastic changes in terms of number and functionality. Usually (but not always) these alterations are reflected in systemic immune alterations. In terms of reproduction and pregnancy, the immune system can be investigated at both local (uterus and decidua) and systemic (peripheral) levels. Investigating the immune system in human decidua is highly restricted because of ethical issues. The evaluation of immune cells and their functions in peripheral blood is a proper alternative [32].

To our knowledge, this is the first study in which the effect of intrauterine administration of autologous PBMCs was investigated on immunologic cells such as Th17, Treg, and NK cells, cytokines, transcription factors, and related miRNAs in humans, pre‐ and post‐treatment.

Many studies evaluated the effect of intrauterine administration of PBMCs, whether activated with hormones or not, on the implantation, pregnancy, live birth, and miscarriage rate in RIF women without considering the alteration of immunologic parameters. A study by Madkour et al. demonstrated that endometrium immuno‐modulation before fresh embryo transfer, by intrauterine insemination of cultured PBMCs, could significantly increase the implantation and clinical pregnancy rate compared to the control group [33]. The same positive effect of PBMC administration for patients with ≥ 3 RIF was observed in the study of Nobijari et al. [34] and Okitsu et al. [35], in which the clinical pregnancy and implantation rate were significantly increased. Systematic reviews and meta‐analyses indicated the benefit of intrauterine PBMCs administration in improving implantation, clinical pregnancy, and live birth in RIF patients [36, 37, 38], in addition to decreasing the miscarriage rate [39]. The study of Pourmoghaddam et al. investigated the effect of intrauterine administration of autologous hCG‐activated PBMCs in the RIF women, considering the patients who had a low Th17, Treg frequency, and Th17/Treg ratio in comparison with the healthy control group before treatment. The results demonstrated that these RIF patients experienced a significantly higher pregnancy and live birth and lower miscarriage rate than the control group, which received PBS; however, the Th17 and Treg cell frequency was not evaluated after treatment. To assess the optimum incubation time for PBMC culture, the culture supernatant was evaluated for cytokine secretion of TNF‐α, IL‐1β, and IFN‐γ and 48 h of culture induced the highest amount of the inflammatory cytokines, in comparison with 2 and 24 h [30].

In the current study, alterations of miRNA expression were also investigated. The results demonstrated that the expression of mir‐10a and mir‐20b‐5p was downregulated post‐treatment. In contrast, the expression of mir‐144‐3p, mir‐155, mir‐718, and mir‐940 was increased following PBMC therapy. According to the literature, mir‐155 targets FoxP3 and PTEN and regulates the expression of PTEN in the HTR8/SVneo cell line as a first‐trimester human trophoblast cell line and, therefore, can regulate apoptosis by the AP‐1/NF‐κB pathway [40]. PTEN may also be involved in the apoptosis of luminal epithelial and decidual cells in mice's decidualization process and trophoblastic cell invasion [19]. The other proposed target for mir‐155 is membrane protein palmitoylated 5 (MPP5) or Stardust, a regulated protein during the menstrual cycle, and its secretion decreases from the proliferative to the late secretory phases [41]. The reduced expression of this protein in RIF patients is consistent with the increased expression of mir‐155 in RIF women [23]. Insulin‐like growth factor 2 (IGF2), a protein expressed in a high amount in the implantation process, is also downregulated by mir‐155 [42]. In addition to mir‐155, mir‐940, downregulated in RIF patients, can also target IGF2 [23]. Another study suggests that mir‐940 expression is significantly upregulated in the placental villi of women who experience miscarriages and can impair trophoblast cells' proliferation. NF‐κB, Wnt/β‐catenin, and ZNF24 are the proposed targets of mir‐940 [20]; however, it is postulated that mir‐940 impairs the proliferation of trophoblast cells and promotes pregnancy wastage by targeting ZNF24 [21]. Therefore, further study needs to be conducted to confirm the exact function of mir‐940. DKK1, a target of mir‐940 and mir‐144‐3p, affects the process of implantation by regulation of embryo attachment [23]. On the other hand, Cox‐2 is negatively regulated by mir‐144‐3p. Downregulation of mir‐144‐3p would lead to Cox‐2 upregulation in preeclamptic placental tissue [43]. Another relevant study also indicated that mir‐144‐3p can target several inflammatory mediators in addition to Cox‐2, including IL‐1β, IL‐6, and TNF‐α, which are involved in the pathogenesis of endometriosis [24]. Additionally, c‐fos, the transcriptional activator of COX2, is the other target of mir‐144, which plays a crucial role in the production of amnion prostaglandin E2 (PGE2) during pregnancy and labor [44] Mir‐718 targets IRAK1 and subsequently negatively regulates NF‐κB activation. On the other hand, PTEN is also targeted by mir‐718. PTEN is capable of controlling the PI3K/Akt pathway. PTEN degradation promotes the phosphorylation of Akt; subsequently, activated Akt suppresses the expression of inflammatory cytokines by NF‐κB [45]. A systemic analysis about the miRNA profile of RIF women indicated that the expression of mir‐718 is downregulated in RIF women [23]. It is suggested that mir‐20b‐5p also affects placental development through regulation of the expression of two proteins, Eph receptor B4 (EPHB4) and ephrin‐B2, which are responsible for trophoblast invasion and angiogenesis [46, 47].

Chen B.Sc. and colleagues' study investigated the differentially expressed miRNAs in RIF patients, which regulate the genes involved in RIF and endometrial receptivity. In line with our findings, the results of the abovementioned study demonstrated that mir‐20b‐5p was upregulated in RIF patients. At the same time, mir‐718, mir‐940, and mir‐144‐3p were downregulated, except mir‐155‐5p, which was upregulated in the Chen B.Sc. study, in contrast with our results [23].

There are controversies about the exact targets and function of miRNAs, from the immunologic point of view, in the process of implantation and pregnancy. The role of some miRNAs is almost confirmed; however, there are disagreements about the others. For example, it has been confirmed that mir‐155 regulates Treg cells' function. Downregulation of this miRNA in RIF patients would lead to overexpression of the targets, such as FoxP3, PTEN, and suppressor of cytokine signaling 1 (SOCS1), known as a negative regulator of cytokine signaling involved in Treg cell suppressor function [11]. Overexpression of FoxP3, PTEN, and probably SOCS1 in RIF patients is modulated by PBMC therapy, as the expression level of mir‐155 was increased and the expression of FoxP3 and PTEN decreased post‐treatment. In addition to mir‐155, mir‐144‐3p, mir‐718, and mir‐940 were also confirmed to be downregulated in RIF patients in relevant studies [23]. However, PBMC therapy was able to elevate the expression level of these miRNAs in our study. According to our data, it is postulated that these four miRNAs are involved in the process of implantation, decidualization, or inflammation and are capable of improving the suitable microenvironment for successful implantation. However, there is some opposite evidence about the anti‐inflammatory effect of mir‐780 and mir‐940.

## 5 Conclusion

Considering the immunomodulatory power of intrauterine PBMC administration for the immunologic aberrations of RIF patients and its positive effects in improving the pregnancy outcome, PBMC therapy may be helpful in treating these patients, especially those with decreased inflammatory cells and mediators in the implantation process. Consistent with this, our data also support the benefit of PBMC administration for regulating the miRNAs involved in the development of reproductive failures, where they mediate the regulation of the immune balance of feto‐maternal crosstalk.

## Conflicts of Interest

The authors declare no conflicts of interest.

## Data Availability

The data that support the findings of this study are available from the corresponding author upon reasonable request.
